# The Role of the Pediatric Yorkhill Malnutrition Score (PYMS), Neutrophil-to-Lymphocyte and Platelet-to-Lymphocyte Ratios in Malnutrition Prediction of Hospitalized Children

**DOI:** 10.3390/children9091378

**Published:** 2022-09-13

**Authors:** Spyridoula Gysi, Michael Doulberis, Corinne Légeret, Henrik Köhler

**Affiliations:** 1 Children’s Hospital of Aarau, 5001 Aarau, Switzerland; 2 Medical Faculty, University of Basel, 4001 Basel, Switzerland; 3 Division of Gastroenterology and Hepatology, Medical University Department, Cantonal Hospital of Aarau, 5001 Aarau, Switzerland; 4 Department of Gastroenterology and Hepatology, University of Zurich, 8091 Zurich, Switzerland; 5 Second Medical Clinic, School of Medicine, Ippokration Hospital, Aristotle University of Thessaloniki, 54652 Thessaloniki, Macedonia, Greece; 6 University Children’s Hospital Basel, 4056 Basel, Switzerland

**Keywords:** malnutrition, pediatric, PYMS, neutrophil, lymphocyte, platelet, ratio, PLR, NLR

## Abstract

Malnutrition in hospitalized children represents a significant burden with occasionally detrimental consequences. In this retrospective analysis of pediatric patients aged one to 16 years old, who were hospitalized in the children’s cantonal hospital of Aarau, Switzerland, we investigated the utilization of PYMS (Pediatric Yorkhill Malnutrition Score) as a routine screening tool for malnutrition in pediatric inpatients. Additionally, we explored the correlation between PYMS and NLR (neutrophil–lymphocyte ratio) and PLR (platelet–lymphocyte ratio), which are two novel biomarkers. Various parameters were analyzed from the medical records of the patients. Most of the sample (n = 211, 77.3%) was characterized by a low PYMS of 0–1 point. Greater NLR and PLR values were significantly associated with greater PYMS (*p* = 0.030 and *p* = 0.004, respectively). ROC (receiver operating characteristic curves) analysis revealed that PLR had a significant predictive ability for having PYMS > 1 (AUC = 0.59; 95% CI: 0.51–0.68; *p* = 0.024). The optimal cut-off was 151 with sensitivity of 51.6% (95% CI: 38.6–64.5%) and specificity of 67.3% (95% CI: 60.5–73.6%). Furthermore, 37% of the children (n = 101) yielded a PLR over 151. Our results support a promising value of PLR as a predictive marker for moderate to severe malnutrition in hospitalized children.

## 1. Introduction

In addition to the underlying disease, hospitalized pediatric patients are jeopardized by developing malnutrition [1,2]. Pediatric malnutrition (undernutrition) has been defined by ASPEN (American Society for Parenteral and Enteral Nutrition) as “*an imbalance between nutrient requirement and intake, resulting in cumulative deficits of energy, protein, or micronutrients that may negatively affect growth, development, and other relevant outcomes*“ [3,4]. Malnutrition as a definition is based on five fundamental characteristics: anthropometric parameters, child’s growth, chronicity of malnutrition, causality with accompanying pathogenesis, as well as functional-developmental outcomes [4].

Pediatric inpatients suffering from acute illness or trauma are often exposed to metabolic stress, and they mostly lack a previous setting of malnutrition. The existence of the inflammatory response within the acute phase of trauma or critical illness and also periods of interrupted feeding contribute to the occurrence of malnutrition [5]. It has to be emphasized that children with malnutrition after discharge are confronted with potentially profound consequences such as failure to thrive and elevated susceptibility to infections [6]. Furthermore, malnutrition has been linked to increased mortality and morbidity as well as hospitalization length and associated health costs [7,8]. Therefore, the identification of such children is of paramount significance.

In this respect, the consensus statement between ASPEN and the Academy of Nutrition and Dietetics recommends the routine malnutrition screening of children upon presentation as well as later throughout their hospitalization [3].

A plethora of screening tools have been developed to identify malnutrition in hospitalized children, with the most prevalent and well-established among them being the Pediatric Yorkhill Malnutrition Score (PYMS), the Screening Tool for the Assessment of Malnutrition in Pediatrics (STAMP), and the Screening Tool for Risk of Impaired Nutritional Status and Growth (STRONG KIDS) [9].

According to the recommendations of the European Society of Clinical Nutrition and Metabolism for nutritional screening, PYMS is a screening tool [10] that has been regularly validated [10,11,12] for evaluating pediatric malnutrition. It was introduced at the Royal Hospital for Sick Children in Yorkhill [10]. The severity of the patient’s nutrition risk is determined by a score that is calculated after four steps that include indicators of malnutrition, such as a history of body weight loss, body mass index (BMI), alterations in nutritional intake, and the impact of acute pathology on nutritional status of the pediatric inpatient [11]. A score of one (1) indicates a medium risk, whereas a score of two (2) or more suggests a high danger of malnutrition [12].

A potential disadvantage of PYMS in real-world clinical practice might be the missing data for the calculation of scores upon a child’s presentation, owing, for instance, to overcrowded emergency departments, loss of awareness of possible underlying malnutrition or disease [13], or patients with scarce communication skills, such as patients with foreign linguistic and cultural backgrounds. Furthermore, several well-known disadvantages, such as overprediction of patients as malnutrition [11] or a learning effect of involved nurses for PYMS application [14], should be mentioned. Therefore, the utilization of values derived from common laboratory findings, such as complete blood counts (CBC), might be a valuable alternative.

In this regard, the neutrophil–lymphocyte ratio (NLR), as well as the platelet–lymphocyte ratio (PLR), represent simple and inexpensive methods for evaluation of a number of pathologies such as acute inflammatory processes, immunosuppression on the ground of transplantation or even psychiatric disorders [15,16,17,18,19,20,21]. Of note, the above-mentioned ratios have been previously utilized with success for the prediction of the nutritional status of geriatric outpatients [22] and adults with chronic obstructive pulmonary disease in intensive care unit settings [23]. In the pediatric population of interest, there is only scarce evidence: According to the accessible literature, Can et al. [16] is the sole study group having investigated NLR and PLR in the analysis of fetal malnutrition neonates.

Within this retrospective study, we aimed primarily to investigate in real-world clinical practice the demographics of hospitalized children, the utilization of PYMS as a routine screening tool, and its role in the follow-up of pediatric inpatients. Additionally, NLR and PLR constitute novel biomarkers utilized as inflammation biomarkers with clinical impact [24] and an emerging role in malnutrition in both pediatric and adult populations [16,23]. Therefore, the secondary aim was the investigation of the correlation between malnutrition (i.e., PYMS) and the above-mentioned indices.

## 2. Patients and Methods

This retrospective study included children aged one to 16 years old, who were hospitalized in the children’s cantonal hospital of Aarau for medical or surgical reasons during the year 2019. For the extraction of medical records, the clinical information system “KISIM” has been utilized (Cistec AG, Zurich, Switzerland). The following parameters have been retrieved from the KISIM medical database; hospitalization length, date of entry, date of birth, gender, diagnosis, body weight, height, BMI, head circumference (percentile und z-score), pediatric Yorkhill malnutrition score (PYMS), co-morbidities, nutritional intervention, platelets, leukocytes and their subsets (neutrophils, lymphocytes), gastrointestinal endoscopies, imaging studies, psychological interventions, request for weight control and other routine laboratory tests recorded in the chart. Curves for z-BMI have been calculated automatically in the clinical information system “KISIM,” which is based on the BMI-WHO (World Health Organization) Growth Charts for boys and girls. The dataset was then extracted to an empty Excel sheet (Microsoft^®^ Excel for Mac 2019, Microsoft Corporation, Redmond, WA, USA) and was further managed with the usage of available Excel filters. The eligibility of the retrieved patients was evaluated by two investigators (S.G. and M.D.), following the inclusion criteria (above-mentioned pediatric ages, available PYMS, and blood samples). Finally, eligible cases were validated by H.K.

## 3. Statistical Analysis

Quantitative variables were expressed as mean ± SD (standard deviation) or as median (interquartile range). Qualitative variables were expressed as absolute and relative frequencies. A Mann–Whitney test was used for the comparison of NLR and PLR between children with PYMS 0–1 and children with PYMS > 1.

To investigate the association of two continuous variables, the Spearman correlation coefficient (rho) has been used. The ROC curve (a receiver operating characteristic) was applied to evaluate the predictability of NLR and PLR. To determine the sensitivity and specificity for optimal cut-offs, the area under the curve (AUC) was computed. All reported *p* values are tailed by two.

Logistic regression analysis was used to investigate whether levels of PLR were associated with having PYMS > 1. Gender, age, and infection were also included in the model. Adjusted odds ratios (OR) with 95% confidence intervals (95% CI) were computed from the results of the logistic regression analyses. Statistical significance was set at *p* 0.05, and analyses were conducted using SPSS Statistics for Windows, version 22.0 (IBM Corp., Armonk, NY, USA).

## 4. Results

A total of 273 children were eligible and considered for our data analysis.

Of this pediatric population, 138 patients were boys (50.5%). The mean age for the whole study population was 7.2 years (4.2 years). The mean BMI was 18 kg/m^2^ (12.5 kg/m^2^), whereas the mean *p* BMI percentile was 50.4% (32%), as well as 13.2% of children were found to have a BMI less than the 10th percentile. Furthermore, the mean duration of hospitalization amounted to 4.2 days (2.5 days). Regarding the disease spectrum, infectious diseases were diagnosed in 151 children (55.1%) of the sample, whereas gastrointestinal diseases amounted to 18 (6.6%). One-third of them (six, 2.2%) had to undergo a gastrointestinal endoscopy. The second most common diagnosis was traumatic head injury, which was mostly mild. No patient with head trauma injury had received anticonvulsant therapy or was in a coma. Cumulative patients’ basic characteristics are illustrated in detail in Table 1.

The median NLR was 2.4 (25th–75th percentile: 1.3–5), and the median PLR was 127.4 (25th–75th percentile: 88.4–180). For further information regarding participants’ biochemical indices and biomarkers, refer to Table 2.

**Figure 1:**
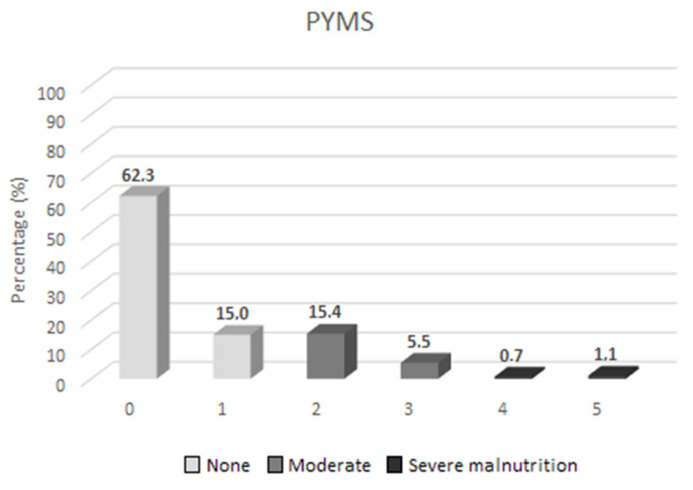
PYMS values; percentage; PYMS 0, 1, 2, 3, 4, and 5.

Most of the sample (n = 211, 77.3%) was characterized by a low PYMS, namely 0 or 1, whereas 57 children (20.9%) were graded as PYMS 2–3, and the rest of the sample (n = 5, 1.8%) was found to have a PYMS of 4 or greater. Overall, 62 (22.7%) of the children had a PYMS of more than one, indicating mild or moderate undernutrition. PYMS values are depicted in Figure 1.

Information about participants’ hospitalization is presented in Table 1, which is stratified by levels of PYMS. The percentage of children being referred to nutritional experts differed significantly (*p* = 0.004) among all three PYMS levels, with the percentage being lower in cases with a PYMS of 0–1. Similarly, the percentage of a nutritionist being present was significantly lower in children with a PYMS of 0–1. Patients with gastrointestinal disease or having undergone a digestive endoscopy were not significantly associated with PYMS (*p* = 0.183 and *p* = 1.000, respectively). Moreover, the percentages of children being referred to a pediatric psychiatrist or having asked to have their weight controlled were significantly greater for children with PYMS values greater than 3 (*p* = 0.014 and *p* = 0.001, respectively).

Furthermore, greater NLR and PLR values were significantly associated with greater PYMS values (*p* = 0.030 and *p* = 0.004, respectively, Table 2). However, NLR and PLR were not significantly associated with BMI percentiles.

**Figure 2:**
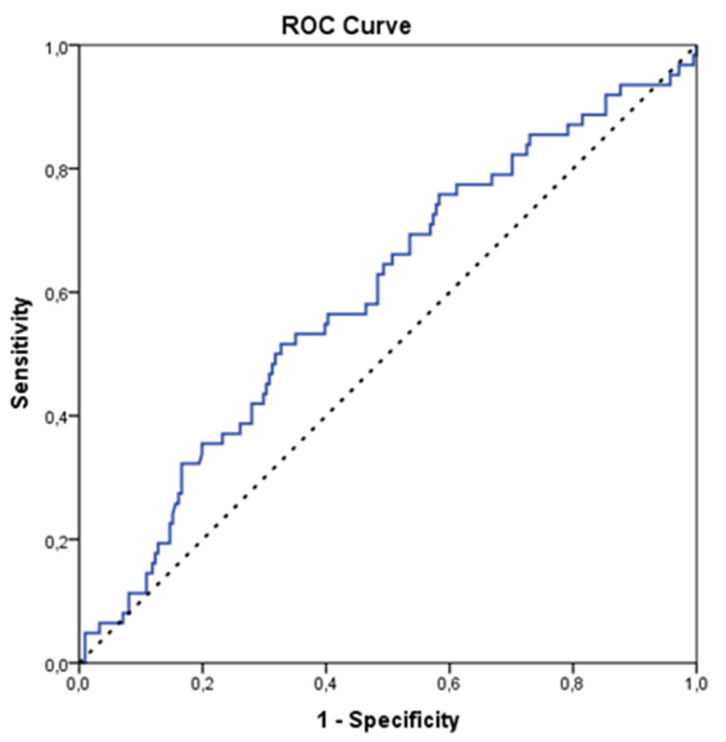
Predictive ability of PLR for having PYMS > 1 according to ROC analysis.

PLR was significantly higher in children with a PYMS of more than one (*p* = 0.024, Table 3). NLR did not differ significantly between children-groups with a PYMS of 0–1 and children with a PYMS of more than one. PLR had a significant predictive ability for having a PYMS > 1 according to ROC analysis (AUC = 0.59; 95% CI: 0.51–0.68; *p* = 0.024) (Figure 2).

The optimal cut-off was 151, and it yielded an estimated sensitivity of 51.6% (95% CI: 38.6–64.5%) and specificity of 67.3% (95% CI: 60.5–73.6%). Furthermore, 37% of the children (n = 101) yielded a PLR of over 151. PYMS > 1 was not significantly predicted by NLP (AUC = 0.52; 95% CI: 0.44–0.51; *p* = 0.580).

Logistic regression revealed that children with a PLR > 151 had a 2.20 times greater probability of having a PYMS > 1 compared to children with a PLR ≤ 151. After adjusting for age, gender, and having an infection, having PLR > 151 remained a significant predictor of having PYMS > 1 (OR = 2.16; 95% CI: 1.21–3.86; *p* = 0.009) (Table 3.)

## 5. Discussion

Within this retrospective study, we screened initially a total of 1043 children being hospitalized in the cantonal hospital of Aarau for the calendar year 2019, and 450 were found to have an available PYMS (43.15%). In this respect, Gerasimidis et al. [10] reported a higher percentage of screened children for PYMS upon admission (72%) and Chourdakis et al. [9] found an even high percentage (86%).

Moreover, in our study, half of the population (50.5%) was boys, which is comparable with yielded percentages of other pediatric malnutrition studies with an inclusion rate of boys 56.9–58% [25]. Furthermore, the calculated mean BMI of this study (18 kg/m^2^) is slightly higher than that of other published studies [25,26]. Regarding the hospitalization duration, the mean 4.2 days of our data parallels the length of stay reported in equivalent studies elsewhere [25,26,27].

Regarding the diagnoses’ spectrum, we found that infectious diseases were the most predominant ones (cumulatively 55.1%), which was followed by traumatic head injury (8.4%) and appendicitis (6.3%). Likewise, Malekiantaghi et al. [26] included in their malnutrition cohort hospitalized children with 43% of the sample consisting of infectious diagnoses and 41% consisting of surgical ones. The frequency of the diagnoses was investigated by Katsagoni et al. [25] in an adequate sample of 1506 children investigating malnutrition in hospitalized patients: gastrointestinal disease (26%), minor surgeries (23.8%), neurological disorders (12.2%), and respiratory disorders (9.5%). It must be stressed, however, that there is only a crude classification of diagnosis as acute or chronic, despite the existence of further pediatric malnutrition investigations for hospitalized children [9,27].

The deviations in findings between our results and the ones of the above-mentioned studies might be explained through possible differences in methodology and characteristics of the included population, such as different genetic or cultural backgrounds.

Considering the fundamental tool of PYMS, most of the sample (77.3%) was characterized by a low PYMS (0–1), whereas 57 children (20.9%) were graded as PYMS 2–3. Only a minority (1.8%) of children had a PYMS greater than 3. Cumulatively, 22.7% of the children had a PYMS greater than one, indicating mild, moderate, or severe undernutrition. These findings contrast with the publication of Gerasimidis et al. [10], where a much lower percentage of about 10% was reported for both children at moderate and elevated risk of malnutrition, as calculated using PYMS. On the contrary, Thomas et al. [28] found in their study that 69.9% of the children had a considerable minimal risk (PYMS 0) for malnutrition, whereas 16% of the total sample was stratified as having medium risk (PYMS 1) and 14% was stratified as having high-risk (PYMS 2), necessitating, therefore, nutritional specialist referral. Furthermore, Chourdakis et al. diagnosed an elevated risk of malnutrition in 25% of the children in a large sample of 2557 children (PYMS, only low and high-risk classification). These discrepancies among the studies might be explained by the diverse set cut-offs for stratification among low, moderate (where included), and elevated risk for malnutrition. A second interpretation might be the relatively small percentage of qualifying children with PYMS completion in our initial screening population (46%). This phenomenon is likely to reflect a further considerable number of children with no malnutrition risk, who were not considered of concern or susceptible to developing malnutrition and therefore did not undergo PYMS upon admission. This non-neglectable “nutritionally healthy” population, if screened with PYMS, might have lowered the percentage of children at minimal risk for malnutrition. Of note, it has to be underlined that although malnutrition occurring after admission to the hospital is not seldom correlated to a risk of adverse clinical events and a longer hospital stay, leading to higher financial burden, it is still a problem that remains underestimated and underreported. Among others, the poor awareness as well as the scarce education of healthcare providers and adverse hospital routines have contributed to this situation [5].

A further substantial novel finding of our study is the calculation of NLR and PLR, which were found to be statistically significantly associated with greater PYMS (*p* = 0.030 and *p* = 0.004, respectively). Additionally, using ROC analysis, we exhibited that PLR had a significant predictive ability for having PYMS > 1 with an optimal cut-off of 151, which is a finding that remained statistically significant (*p* = 0.009) even after adjusting for potential confounders (age, gender, and having an infection).

According to the literature we have access to, this is the first study to link these inflammatory biomarkers with validated screening equipment (PYMS) for pediatric inpatient malnutrition. In a neonatology study, Can et al. [16] investigated the relationship between NLR, PLR, and neonatal malnutrition. The investigators recruited a large-scale singleton term appropriate for gestational age neonates (n = 4320), which were evaluated for malnutrition. Of these, 6% were found to be malnourished. Moreover, in correlation analyses, there was a negative association between PLR as well as NLR with fetal nutritional status (*p* = 0.001 and *p* = 0.011, respectively).

Further pediatric studies outside the spectrum of malnutrition, albeit under the prism of PLR and NLR, have been previously published, particularly regarding appendicitis, transplanted children, or even psychiatric disorders [15,16,17,18,19,20,21].

Concerning adult scientific evidence of PLR, NLR, and malnutrition, the scarce existing literature confines itself to the following studies: Kaya et al. [22] screened 95 geriatric patients (age > 65 years), and the nutritional status of included individuals was assessed using the Mini Nutritional Assessment and Geriatric Nutritional Risk Index. Further estimations included albumin, total cholesterol, body mass index, mid-arm circumference, and calf circumference. Finally, NLR was assessed by using the available CBC. The authors concluded, after performing sensitivity analyses as well as logistic regression, that NLR was an independent factor for the estimation of malnutrition or risk of malnutrition in senior patients. A second adult and very recent study utilizing the ratios of interest stems from Baldemir et al. [23]. The authors included patients with chronic obstructive pulmonary disease hospitalized in intensive care units. The key questions were about an association between prognostic nutritional index (PNI), nutritional risk screening (2002), and nutrition risk in the critical illness (Nutric) Score and determining a cut-off value for PNI, PLR, NLR, and lymphocyte-to-monocyte ratio. They concluded that NLR, PLR, LMR, basophil, and lymphocyte values may be useful in the evaluation of the nutritional status of such patients.

NLR and PLR are considered acknowledged inflammatory markers [24]. Inflammation as the key factor has been previously linked to the cascade of the malnutrition–inflammation–atherosclerosis and calcification syndrome [29]. In addition, the raise of platelets and neutrophils but the fall of lymphocytes represent several of the hematological hallmarks in children diagnosed with severe acute malnutrition [30]. Moreover, BMI status has been also demonstrated to be a common denominator significantly affecting blood cell counts, i.e., predominantly lymphocyte, neutrophil, and platelet counts [31].

Particularly regarding PLR, it has been established as a useful marker indicating inflammatory shifts of lymphocyte and platelet counts. PLR has been thoroughly investigated in oncologic, transplanted as well as hematologic and rheumatologic patients. The significance of PLR as an inflammatory marker is exceptional, once its variations are considered together with other complementary indices, especially NLR, which yields further useful information regarding the activity of disease or inflammation. PLR is believed to better predict the clinical outcomes of individuals with systemic inflammation compared to other counts (i.e., platelet or lymphocyte). The extent of stress-induced hypercortisolemia with a secondary release of platelets into the bloodstream and transient lymphopenia affect the degree of PLR rise across a plethora of proinflammatory and prothrombotic disease states. Such a non-specific pathogenetic cascade of PLR increase might be counteracted by intensified platelet destruction or consumption at the sites of immune inflammation and thrombosis, requiring cross-checks of all blood cell counts and various inflammatory and immune markers [32].

Despite the previously mentioned novelties and findings, our study is characterized by several limitations: namely, the retrospective nature of the study design as well as the relatively small sample have to be acknowledged. In addition, an association does not imply causality. Furthermore, confounding effects of glucocorticoids might affect CBC, and a single “snapshot” of CBC does not necessarily reflect their dynamics. Additionally, the specificity of the shifts in PLR is rarely neglected in individuals with chronic comorbidities, such as metabolic, vascular, autoimmune, and neoplastic ones. It has also to be emphasized that those molecular pathways of activation of platelets and the rest of the blood cells were not estimated to unravel the associations to their full extent with cellular markers of inflammation. In addition, there are no up-to-date valid age-, ethnic-, and gender official recommendations for the usage of CBC [32]. Lastly, but most importantly, our findings necessitate further substantiation. In this respect, further large-scale, ideally prospective, studies with parallel malnutrition scores and defined validation criteria are warranted before such an inexpensive and practical predictive ratio finds a place in routine clinical pediatrics.

## 6. Conclusions

Pediatric malnutrition, especially in inpatient children, represents a serious and often underestimated medical problem, which may conceal long-term detrimental consequences if not early diagnosed and correctly treated. PYMS remains one of the most validated and reliable screening tools for children with or jeopardizing to the development of undernutrition (malnutrition), which is suggested to be performed routinely on all patients upon admission. Nevertheless, the occasional lack of awareness or the insufficient training of health care providers, the crowded emergency departments, or further potential difficulties such as linguistic and cultural particularities of referred children, may limit its utilization. PLR and NLR are regarded as practical and easy-to-perform indices which have been previously well-established for adult and pediatric clinical outcomes, including inflammatory and immunomodulatory ones. Our results support a promising value of PLR as a predictive marker for moderate to severe malnutrition in children. Further large-scale relevant pediatric studies are highly encouraged in order for the usefulness and validity of this simple marker to be further clarified.

## Figures and Tables

**Table 1:** (a) Participants’ characteristics, (b) information according to PYMS.

*a. Participants’ Characteristics*				
Variable	N (%)			
Gender				
Males	138 (50.5)			
Females	135 (49.5)			
Age in years, mean (SD)	7.2 (4.2)			
BMI mean (SD) 18 (12.5)				
P.BMI mean (SD) 50.4 (32.9)				
P.BMI < 10 kg/m^2^	34 (13.2)			
Days of hospitalization, mean (SD) 4.2 (2.5)				
***b. Participants’ information regarding their hospitalization according to PYMS***				
Variable	PYMS			P+
	0 to 1	2 to 3	≥4	
	N (%)	N (%)	N (%)	
Referral to nutritional experts				
	207 (98.6)	52 (91.2)	4 (80.0)	0.004
Yes	3 (1.4)	5 (8.8)	1 (20.0)	
Pre-existing nutritional support				
No	207 (98.1)		4 (80.0)	0.009
Yes	4 (1.9)		1 (20.0)	
Gastrointestinal disease				
No	199 (94.3)	52 (91.2)	4 (80.0)	0.183
			1 (20.0)	
Endoscopy				
No	206 (97.6)	56 (98.2)	5 (100.0)	1000
		1 (1.8)	0 (0.0)	
Referral to psychiatrist/psychologist				
No	203 (96.7)	54 (94.7)	3 (60.0)	0.014
Yes	7 (3.3)	3 (5.3)	2 (40.0)	
Weight control–outpatient follow-up				
No	208 (98.6)	52 (91.2)	3 (60.0)	<0.001
	3 (1.4)	5 (8.8)	2 (40.0)	

*p* BMI, Percentile body mass index (for age); SD, standard deviation, +Fisher’s exact test, PYMS, Pediatric Yorkhill malnutrition score.

**Table 2:** (a) Participants’ biochemical indexes and biomarkers, (b) Spearman correlation coefficient of NLR and PLR with PYMS and BMI percentiles.

*a. Participants’ Biochemical Indexes and Biomarkers*			
	Mean (SD)	Median (IQR)	
Platelets	306.9 (104)	297 (248–358)	
Leukocytes	11.2 (5.9)	9.9 (7.3–13.6)	
Neutrophils	7.4 (5.6)	5.9 (3.9–9.4)	
Lymphocytes	2.6 (1.6)	2.4 (1.6–3.3)	
NLR	4.3 (5.2)	2.4 (1.3–5)	
*PLR*			
***b. Spearman correlation coefficient of NLR and PLR with PYMS and BMI percentiles***			
		PYMS	*p* BMI
NLR	rho	0.13	−0.03
		0.030	0.594
PLR	rho	0.17	0.00
	*p*	0.004	0.964

IQR, interquartile range; NLR, neutrophil to lymphocyte ratio; PLR, platelet to lymphocyte ratio; SD, standard deviation NLR, neutrophil to lymphocyte ratio; *p* BMI, percentile body mass index (for age); PLR, platelets to lymphocyte ratio, PYMS, pediatric Yorkhill malnutrition score.

**Table 3:** (a) NLR and PLR values according to PYMS, (b) the odds ratio of PLR levels for the prediction of having a PYMS > 1.

*a. NLR and PLR values according to PYMS*					
	PYMS				*p*+
	0–1	>1			
	Mean (SD)	Median (IQR)	Mean (SD)	Median (IQR)	
NLR	4.41 (5.57)	2.4 (1.31–4.51)	3.92 (3.56)	2.83 (1.33–5.51)	0.58
PLR	151.99 (112.23)	123.72 (86.62–176.97)	177.22 (122.02)	153.22 (107.42–211.11)	0.024
***b. the odds ratio of PLR levels for the prediction of having PYMS > 1***					
	OR (95% CI) +	*p*	OR (95% CI) ++	*p*	
PLR					
≤151 (Reference)					
>151		0.007	2.16 (1.21–3.86)	0.009	

*p*+ Mann–Whitney test, IQR, interquartile range; NLR, neutrophil to lymphocyte ratio; PLR, platelets to lymphocyte ratio; PYMS, pediatric Yorkhill malnutrition score; OR, odds ration, SD, standard deviation; + Crude OR; ++ OR adjusted for age, gender and having infection.

## Data Availability

Data extracted from medical records of the clinical information system “KISIM” (Cistec AG, Zurich, Switzerland).

## Abbreviations

ASPEN, American society for parenteral and enteral nutrition; AUC, area under the curve; BMI, body mass index; CBC, cell blood counts; NLR, neutrophil–lymphocyte ratio; PLR, platelet–lymphocyte ratio; PYMS, pediatric Yorkhill malnutrition score; ROC, receiver operating characteristic (curves); SD, standard deviation.
